# Anti-tumor effects of everolimus and metformin are complementary and glucose-dependent in breast cancer cells

**DOI:** 10.1186/s12885-017-3230-8

**Published:** 2017-03-29

**Authors:** Gerke Ariaans, Mathilde Jalving, Emma Geertruida Elisabeth de Vries, Steven de Jong

**Affiliations:** Department of Medical Oncology, Cancer Research Center Groningen, University of Groningen, University Medical Center Groningen, Hanzeplein 1, 9713 GZ Groningen, The Netherlands

**Keywords:** Metformin, Everolimus, Glycolysis, Hypoxia, Breast cancer, Metabolism

## Abstract

**Background:**

Clinical efficacy of the mTOR inhibitor everolimus is limited in breast cancer and regularly leads to side-effects including hyperglycemia. The AMPK inhibitor and anti-diabetic drug metformin may counteract everolimus-induced hyperglycemia, as well as enhancing anti-cancer efficacy. We investigated the glucose-dependent growth-inhibitory properties of everolimus, metformin and the combination in breast cancer cell lines.

**Methods:**

The breast cancer cell lines MCF-7, MDA-MB-231 and T47D were cultured in media containing 11 mM or 2.75 mM glucose with 21% or 1% oxygen. Everolimus and metformin treated cells were subjected to cytotoxicity and clonogenic assays, western blotting, FACS and metabolic measurements.

**Results:**

Everolimus was less effective in MCF7 cells under low glucose conditions compared to high glucose conditions (IC_50_ of >50 nM vs 29.1 ± 1.4 nM) in a short-term survival assay, while sensitivity of MDA-MB-231 and T47D cells to everolimus was lost under low glucose conditions. In contrast, metformin was more effective in low than in high glucose conditions in MCF7 (IC_50_ of 1.8 ± 1.2 mM vs >5 mM) and MDA-MB231 cells (1.5 ± 1.3 mM vs 2.6 ± 1.2 mM). Metformin sensitivity of T47D cells was independent of glucose concentrations. Everolimus combined with metformin additively inhibited cell survival, clonogenicity, mTOR signaling activity and mitochondrial respiration. These effects were not the result of enhanced autophagy or apoptosis induction. Similar results were observed under hypoxic conditions.

**Conclusion:**

Metformin-induced effects are additive to the anti-proliferative and colony inhibitory properties of everolimus through inhibition of mitochondrial respiration and mTOR signaling. These results warrant further in vivo investigation of everolimus combined with metformin as a putative anti-cancer therapy.

**Electronic supplementary material:**

The online version of this article (doi:10.1186/s12885-017-3230-8) contains supplementary material, which is available to authorized users.

## Background

The mammalian target of rapamycin (mTOR) pathway, hyperactive in numerous cancer types including breast cancer, is an attractive therapeutic target. Disappointingly, mTOR inhibitors only show clinical benefit in selected settings and efficacy is limited. Moreover, toxicity, including fatigue and mucositis limit clinical use [1]. mTOR signaling is central in the integration of cellular signals involved in growth and cellular energy status [2]. Therefore, the metabolic context of mTOR inhibition in cancer cells is essential for understanding and improving its anti-tumor effects and toxicity profile.

The mTOR protein is the catalytic subunit of two structurally and functionally different protein complexes: mTORC1 and mTORC2. mTOR complex 1 (mTORC1) is sensitive to growth factor signaling, oxygen levels and nutrient availability. Downstream, mTORC1 inhibits the transcriptional repressor eukaryotic initiation factor 4B binding protein (4EBP1), and activates S6 ribosomal protein (S6), leading to expression of proteins essential for the regulation of cell growth. mTOR complex 2 (mTORC2) regulates AKT activity through phosphorylation and is involved in cell survival and proliferation. Moreover, mTORC2 induces expression of glycolytic enzymes, pentose phosphate pathway enzymes and glutaminase and increases cellular lipogenesis [3]. Everolimus, the most commonly used mTOR inhibitor, directly inhibits mTORC1, but also (indirectly) inhibits mTORC2 [4, 5]. This mTORC2 inhibition may underlie the induction of hyperglycemia in a large proportion of patients treated with everolimus [6, 7]. High glucose levels can stimulate tumor growth in patients and are associated with resistance to breast cancer chemotherapy [8, 9]. It is currently unknown whether hyperglycemia counteracts anti-proliferative effects of everolimus. Cancer patients on everolimus treatment are regularly treated with anti-diabetic drugs, especially metformin, to reduce glucose levels. Metformin is a widely prescribed, well-tolerated, effective treatment for type 2 diabetes mellitus. Moreover, epidemiological evidence and retrospective clinical data indicate, that metformin has intrinsic anti-cancer properties [10, 11]. At the cellular level, metformin inhibits complex I of the mitochondrial respiratory chain leading to compensatory increases in glycolytic flux and activated AMP-activated kinase (AMPK) [12]. This results in growth inhibition of tumor cells through inhibition of mTOR, cell cycle arrest, activation of autophagy and possibly apoptosis [13]. Thus, everolimus and metformin both inhibit mTOR signaling and, moreover, differentially target tumor cell glucose metabolism.

We hypothesized that the combination of everolimus and metformin would synergistically inhibit cell growth in a glucose concentration dependent manner. To test this hypothesis and predict potential clinical value of the combination, culture conditions optimally reflecting in-vivo tumor metabolic circumstances are required. Strikingly, in most in vitro studies, media containing up to 25 mM glucose are used. This is 4–5-fold higher than the mean fasting blood serum glucose levels of healthy individuals. Additionally, poorly vascularized areas of tumors may have even lower glucose concentrations and hypoxia may be present. In the present study, we therefore investigated the growth inhibitory effects and underlying signal transduction and metabolic mechanisms of everolimus and metformin treatment alone, and in combination, at physiological glucose concentrations in hypoxic and normoxic conditions in breast cancer cell lines.

## Methods

### Reagents and cell culture

Everolimus (Sigma-Aldrich, Zwijndrecht, The Netherlands) was dissolved in dimethyl sulfoxide (DMSO) to a concentration of 20 mM and diluted in phosphate buffered saline (PBS, 0.14 M NaCl, 2.7 mM KCl, 6.4 mM Na_2_HPO_4_.2H_2_O, 1.5 mM KH_2_PO_4_, pH 7.2–7.5) prior to use. Metformin (Sigma-Aldrich, Zwijndrecht, The Netherlands) was dissolved to a concentration of 1 M in PBS and stored at −20 °C until use. The human tumor cell lines used were purchased from the American Type Culture Collection (ATCC, Manassas, USA). The luminal A MCF-7 (catalog number HTB-22) and luminal A T47D (catalog number HTB-133) breast cancer cells were cultured in RPMI containing 11 mM glucose, supplemented with 10% FCS at 37 °C in 5% CO_2_. Triple negative MDA-MB-231 breast cancer cells (catalog number HTB-26) [14] were cultured in DMEM containing 11 mM glucose, supplemented with 10% fetal calf serum (FCS) and 1 mM glutamine at 37 °C in 5% CO_2_. Cultures in 5.5 mM glucose were maintained by adding the appropriate amount of glucose-free RPMI/DMEM to standard RPMI (all Gibco Thermo Fisher Scientific, Bleiswijk, The Netherlands). Glucose concentrations in cell culture media were measured using the Accu-Chek Aviva glucose meter (Roche, Almere, The Netherlands). Accuracy of measurements of glucose concentrations in cell culture media was confirmed using a calibration curve constructed using fresh culture medium with known glucose concentrations. The detection limit of the Accu-Check is 0.6 mM glucose. Experiments using 2.75 mM glucose in the cell culture media were performed using cells that were cultured in 5.5 mM glucose and were prepared in 2.75 mM glucose containing medium 24 h before the start of the experiment. For hypoxia experiments, cells were placed in an incubator with 1% oxygen and 5% CO_2_ after the addition of reagents.

### Viability assay and colony survival assay

For the viability assays MCF7, T47D and MDA-MB-231 cells were plated at a density of 2000, 2500 or 3000 cells per well, respectively, in 96 wells plates (4 wells/condition) and subsequently incubated with metformin and everolimus at the desired concentrations for 4 days in the same culture medium, that was also used for cell culture. For MCF7 and T47D RPMI-media containing 11 or 2.75 mM glucose was used. For MDA-MB-231 DMEM containing 11 or 2.75 mM glucose was used. After 4 days 20 μl 3-(4,5-dimethylthiazol-2-yl)-2,5-diphenyltetrazolium bromide solution (5 mg/ml in PBS) was added to each well. After 4 h of incubation formazan crystals were dissolved in 200 μl DMSO and absorption at 520 nm wavelength was determined with a plate reader (iMark, BioRad, Veenendaal, The Netherlands). No major effects of metformin on the relationship between cell numbers and MTT conversion were observed. For each experiment MTT results were visually checked by light microscopy. For the colony survival assay cells were plated in 6-wells plates. 250 cells/well were plated and allowed to adhere for at least one hour before treatment. When glucose was replenished, 2.75 mM glucose was added every other day for in total 3 times to achieve a total amount of usable glucose of 11 mM during the course of the experiment. Pilot data demonstrated that this procedure ensured the presence of relatively stable glucose levels during the course of the drug treatment. After 8 days of treatment, cells were fixed and stained with Coomassie blue. Colonies consisting of at least 50 cells were counted.

### Western blotting analysis

MCF-7 and MDA-MB-231 cells were lyzed in MPER (Thermo Scientific, Bleiswijk, The Netherlands) and diluted 1:1 with SDS sample buffer (4% SDS, 20% glycerol, 0.5 mol/l Tris-HCl (pH 6.8), 0.002% bromophenol blue). Lysates were resolved by SDS-PAGE and transferred to PVDF membranes. Membranes were incubated overnight at 4 °C and probed with the following antibodies: rabbit-anti-AKT, rabbit-anti-pAKT (Thr308), rabbit-anti-S6, rabbit-anti-pS6, rabbit-anti-4EBP1 (all Cell Signaling Technologies, Leiden, The Netherlands) in a 1:1000 dilution or anti-HIF1α (BD Biosciences, Breda, The Netherlands) and mouse-anti-actin (MP Biomedicals, Santa Ana, USA) in a 1:10,000 dilution. Primary antibodies were stained using HRP-coupled goat anti-rabbit or rabbit anti-mouse IgG and developed with Lumi-Light (Roche, Almere, The Netherlands). Images were captured with the ChemiDoc MP imaging system (Bio-Rad, Veenendaal, The Netherlands) and Image Lab Software.

### Quantification of autophagy, reactive oxygen species (ROS), and cell death

MCF-7 and MDA-MB-231 cells were transfected with a GFP-LC3 containing retrovirus (kindly provided and developed by H Folkerts, Department of Experimental Hematology, University Medical Centre Groningen, the Netherlands). Upon upregulation of autophagy the LC3-GFP protein forms aggregates that can be visualized using fluorescence microscopy. Bafilomycin A1, a known inhibitor of the late phase of autophagy, efficiently blocks turnover of autophagic vesicles, thereby increasing LC3-GFP foci. GFP-LC3 expressing MCF-7 and MDA-MB-231 were grown on cover slips and treated with metformin, everolimus and 20 nM bafilomycin (Sigma-Aldrich, Zwijndrecht, The Netherlands) for the indicated duration. Cells were washed with cold PBS and fixed with 3.7% paraformaldehyde. Cover slips were mounted on glass plates using Kaiser’s mounting medium. Fluorescent GFP-LC3 foci per individual cell were counted. Moreover, cleavage of the LC3 protein was determined using Western Blotting with an anti-LC3 antibody (Cell Signaling Technology, Leiden, The Netherlands). ROS measurement was performed using H_2_DCF (Sigma-Aldrich, Zwijndrecht, The Netherlands). Hydrogen peroxide treated cells were used as a positive control. After harvesting by trypsinization, cells were washed once with PBS and subsequently incubated with 10 μM H_2_DCF for 30 min at 37 °C. Samples were washed with cold PBS and analyzed using a FACSCalibur (Becton Dickinson, Breda, The Netherlands). Analysis was performed using Flowing software 2.5 (Informer Technologies, Inc).

Four days prior to cell death measurements, cells were plated at the desired density, treated with metformin and everolimus and supplemented with 2.75 mM glucose (1 M stock solution) each day. On the day of analysis, cells were harvested by trypsinization and washed once in calcium-buffer. Cells were subsequently incubated in a 1:12 dilution of annexin V-FITC antibody (IQ products, Groningen, The Netherlands) in calcium buffer for 20 min on ice. Samples were washed with calcium-buffer and resuspended in calcium-buffer containing 0.5 μg/ml propidium iodide (PI). Cells were analyzed immediately using a FACSCalibur (Becton Dickinson, Breda, The Netherlands). Analysis was performed using Flowing Software 2.

### Quantification analyses of mitochondrial respiration and glycolysis

Mitochondrial and glycolytic function of MCF7 and MDA-MB-231 cells was determined using a Seahorse XF24 Extracellular Flux Analyzer (Seahorse Bioscience, North Billerica, USA). Cells were seeded with an appropriate density in specialized V7 Seahorse tissue culture plates (3 wells/condition). After 2 days cells were treated with indicated concentrations of metformin, everolimus or a combination and incubated for another 2 days. On the day of the measurements, cells were washed once with PBS and once with unbuffered 1 mM sodium pyruvate containing XF assay medium (pH 7.4) and 11 mM or 2.75 mM glucose, respectively. The assay commenced after cells had been incubated in 500 μl unbuffered XF assay medium (pH 7.4) for 1 h. Baseline oxygen consumption rate (OCR) and extracellular acidification rate (ECAR) were determined. To gather detailed information about the mitochondrial and glycolytic function of the cell lines MCF-7 and MDA-MB-231 in response to treatment with metformin and everolimus a mitochondrial stress test was performed. Using the ATP-synthase inhibitor oligomycin, the mitochondrial uncoupler carbonyl cyanide 4-(trifluoromethoxy)phenylhydrazone (FCCP) the complex I inhibitor rotenone and the cytochrome C reductase inhibitor antimycin A (all Sigma-Aldrich, Zwijndrecht, The Netherlands) a detailed profile of basal respiration, maximal respiration and induction of glycolysis can be gathered. Three technical replicates were performed per sample. OCR and ECAR were normalized for the amount of cellular protein in each well using the seahorse XF24 software. Protein amount was determined using the Bradford assay. The three measurements of each step of this mitochondrial stress test were combined for analysis.

### Statistical analyses

Data are presented as mean ± standard deviation (SD). Different experimental conditions were compared using unpaired Student’s t-tests. Statistical analyses were performed using Prism v.5 (GraphPad). A *P*-value of <0.05 (two-tailed) was considered significant.

## Results

### Inhibition of cell viability by everolimus and metformin is glucose-dependent

**Fig. 1:**
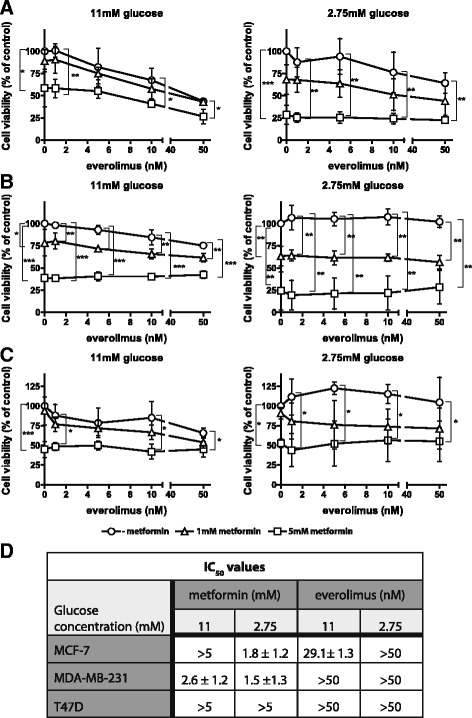
Inhibition of cell viability by everolimus and metformin is glucose-dependent MCF-7 (**a**), MDA-MB-231 (**b**) and T47D (**c**) cells cultured in 11 mM or 2.75 mM glucose-containing medium were treated with everolimus (1-50 nM) and/or metformin (1 and 5 mM) for 96 h. Cell viability was measured using an MTT assay. IC_50_-values were calculated for everolimus and metformin in high and low glucose conditions (**d**). Data are presented as mean ± SD of three different experiments. * *p* < 0.05; ** *p* < 0.01; *** *p* < 0.001; *n* = 3

In order to determine whether glucose levels have an effect on the inhibition of cell viability by everolimus and metformin in a short-term treatment setting of 4 days, an MTT-based cell survival assay was carried out. Everolimus inhibited viability of luminal A wild-type p53 MCF7 breast cancer cells, and to a lesser extent viability of the triple negative mutant p53 MDA-MB-231, and luminal A mutant p53 T47D breast cancer cells in culture medium containing 11 mM glucose. In culture medium containing 2.75 mM glucose, effects of everolimus on cell viability were reduced for MCF7 cells and completely lost for MDA-MB-231 and T47D cells (Fig. 1a-d). Metformin inhibited viability of all three cell lines in a concentration dependent manner at both glucose concentrations. Metformin treatment of MCF7 and MDA-MB-231 cells was more effective in 2.75 mM glucose medium than in 11 mM glucose medium (Fig. 1a-d). The effects of everolimus and metformin in combination treatment were additive in both high and low glucose conditions. Since everolimus was less effective but metformin more effective in low glucose conditions, the overall effect of combination remained the same in high and low glucose conditions (Fig. 1a-d).

The majority of luminal A breast cancers are wild-type p53 and triple negative breast cancer are frequently mutant p53. Therefore, we used MCF7 and MDA-MB231 as representatives of these breast cancer subtypes for further analyses.

### mTOR inhibition by metformin is dependent on glucose concentration

**Fig. 2:**
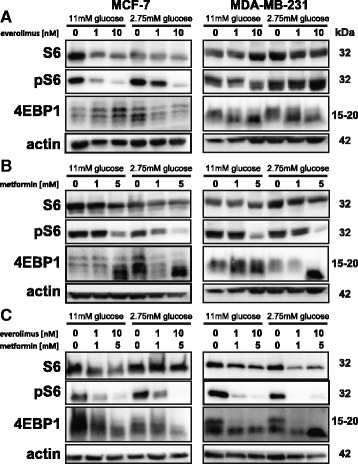
Effect of glucose concentration on inhibition of mTOR signalling by everolimus and metformin. Western Blotting analysis was carried out for S6, pS6 and 4EBP1. Cells were treated for 2 days with indicated concentrations of metformin (**a**), everolimus (**b**) or a combination (**c**) Representative Western Blots are shown

Subsequently, we examined the ability of everolimus and metformin to inhibit mTOR signaling in MCF-7 and MDA-MB-231 cells in the presence of high and low glucose concentrations using p-S6 levels as read-out for mTOR signaling. Everolimus inhibited the mTOR signaling pathway in a concentration dependent fashion at high glucose concentration in MCF7 cells but only marginally in MDA-MB-231 cells, as demonstrated by the reduction in p-S6 (Fig. 2a). The effect of everolimus (1 and 10 nM) on p-S6 levels at low glucose concentration was reduced in MCF7 cells and lost in MDA-MB-231 cells (Fig. 2a). These results are in agreement with the cell viability assay results (Fig. 1a and b). Metformin (1 and 5 mM) also inhibited the mTOR-signaling pathway in both MCF-7 and MDA-MB-231 cells in a drug concentration dependent manner as indicated by reduced p-S6 levels (Fig. 2b). Metformin more effectively diminished p-S6 levels in both cell lines at low glucose concentration in concurrence with the viability assay results. The combination of everolimus and metformin resulted in a strong inhibition of the mTOR pathway at both glucose concentrations in MCF-7 and MDA-MB-231 cells (Fig. 2c).

As a second read-out for mTOR signaling we used 4EBP1. The lower molecular weight bands probably reflect less phosphorylated 4EBP1, indicating stronger inhibition of cap-dependent initiation of mRNA translation by 4EBP1 [15]. A shift in 4EBP1 band intensity from the higher to the lower molecular weight band was observed in both cell lines treated with everolimus (Fig. 2a). Metformin also induced a concentration dependent band shift of 4EBP1 in MCF-7 and MDA-MB-231 cells (Fig. 2b). The combination of everolimus and metformin resulted in a band shift of 4EBP1, independently of glucose concentration (Fig. 2c). Taken together, these results suggest an additive effect of everolimus and metformin on mTOR pathway inhibition.

### Everolimus and metformin do not alter autophagy levels or ROS formation

**Fig. 3:**
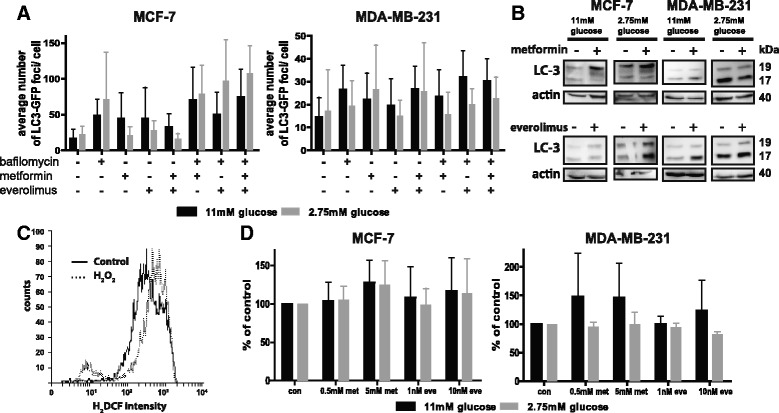
Metformin and everolimus do not influence autophagy and ROS production. **a** MCF-7 and MDA-MB-231 cells stably expressing LC3-GFP cultured in 11 mM and 2.75 mM glucose-containing medium were treated with indicated concentrations of 10 nM everolimus and/ or 5 mM metformin for 48 h. 20 μM bafilomycin was added 1 h prior to evaluation of the assay. Fluorescent GFP-LC-3 foci per individual cell were counted. Data are presented as mean ± SD of three different experiments. **b** MCF-7 and MDA-MB-231 cells cultured in 11 mM and 2.75 mM glucose-containing medium were treated with everolimus (10 nM) or metformin (5 mM) for 48 h. Western Blotting for LC-3 revealed that there is no increased LC-3 cleavage after either treatment. **c** MCF-7 cells were treated with 250 μM H_2_O_2_ for 1 h. Hydrogenperoxide induces ROS production in MCF-7 cells, as demonstrated by increase in cellular H_2_DCF fluorescence. **d** MCF-7 and MDA-MB-231 cultured in 11 mM or 2.75 mM glucose-containing medium were treated with indicated concentrations of everolimus or metformin for 48 h, and stained with 10 μM H_2_DCF. Everolimus and metformin do not induce significant changes in ROS levels compared to untreated cells. Data are presented as mean ± SD of three different experiments

Previous research suggests that everolimus and metformin are able to induce autophagy [16]. However, treatment with everolimus, metformin, or a combination of both drugs did not induce changes in LC3-GFP foci formation in MCF7 and MDA-MB-231 cells (Fig. 3a). In addition, Western blotting of cleaved LC-3 did not show increased autophagy due to everolimus and/or metformin treatment at any glucose concentration (Fig. 3b). LC-3 cleavage was not influenced by oxygen tension (data not shown). These results indicate that everolimus and metformin did not interfere in autophagic processes in these models.

Due to the inhibition of complex I in mitochondria by metformin, we expected a decrease in the formation of ROS [17]. Everolimus, metformin or both drugs combined did not affect ROS formation at the indicated concentrations in MCF7 and MDA-MB-231 cells. In contrast, treatment with the known ROS inducer hydrogen peroxide led to an increase in cellular H_2_DCF fluorescence (Fig. 3c-d).

### Everolimus reduces mitochondrial respiration, whereas metformin also increases glycolysis

**Fig. 4:**
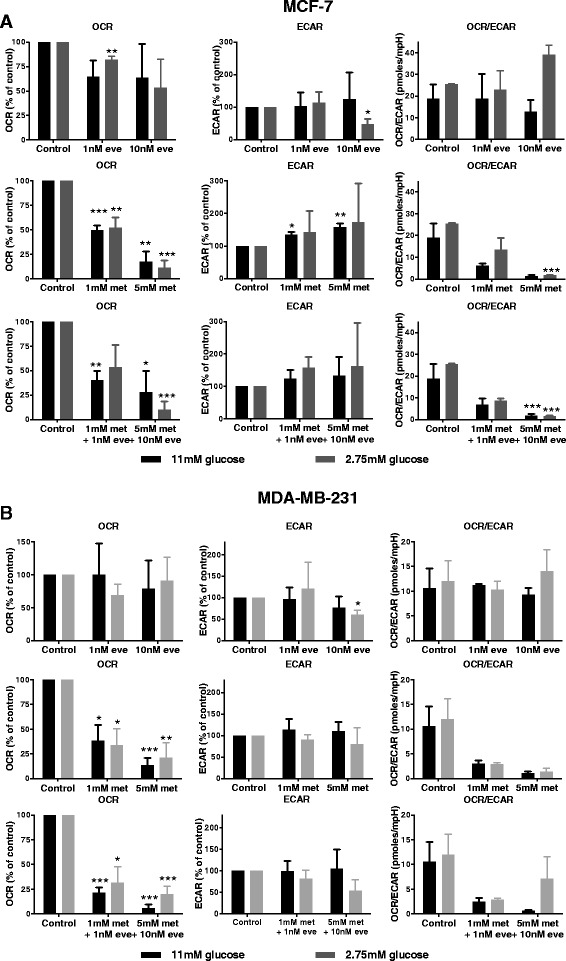
Oxygen consumption and extracellular acidification after treatment with metformin and everolimus for 48 h. Using the seahorse XF analyzer, the OCR, the ECAR and the ratio of both parameters of MCF-7 (**a**) and MDA-MB-231 cells (**b**) in response to 48 h of metformin or everolimus treatment was determined. Data are presented as mean ± SEM of three different experiments. Treated samples were compared to the same glucose concentration control. * *p* < 0.05; ** *p* < 0.01; *** *p* < 0.001; *n* = 3

Figure 4 shows the effects of everolimus and metformin on mitochondrial respiration (OCR) and glycolysis (ECAR) in MCF7 and MDA-MB-231 cells. Basal OCR did not significantly decrease when cells were treated with everolimus. In agreement, everolimus did not induce changes in ECAR. Metformin dose-dependently reduced basal OCR in MCF7 and MDA-MB-231 cells. In contrast to everolimus treatment, metformin also dose-dependently increased basal ECAR of MCF7 cells, indicating an induction of glycolytic processes in response to metformin treatment. This shift from mitochondrial respiration to glycolysis in MCF7 cells was most pronounced in high glucose media. A combination of everolimus and metformin strongly reduced the OCR/ECAR ratio, even when low doses were used. However, there is no difference in OCR/ECAR ratio between metformin treated cells and combination treated cells suggesting a dominant effect of metformin on cell metabolism. Another important difference between the effect of metformin and everolimus is that uncoupling of the mitochondrial respiration with FCCP in metformin treated cells resulted in an enhancement of respiration (Additional file 1: Figure S3). Respiration levels, however, were still lower than respiration levels in untreated cells after uncoupling of mitochondrial respiration. Uncoupling with FCCP did not elevate mitochondrial respiration in everolimus treated cells. This suggests that metformin treatment inhibits mitochondrial respiration and partially reduced the mitochondrial capacity, whereas everolimus treatment resulted in a loss of mitochondrial capacity.

### Metformin does not induce cell death under stable low glucose conditions

**Fig. 5:**
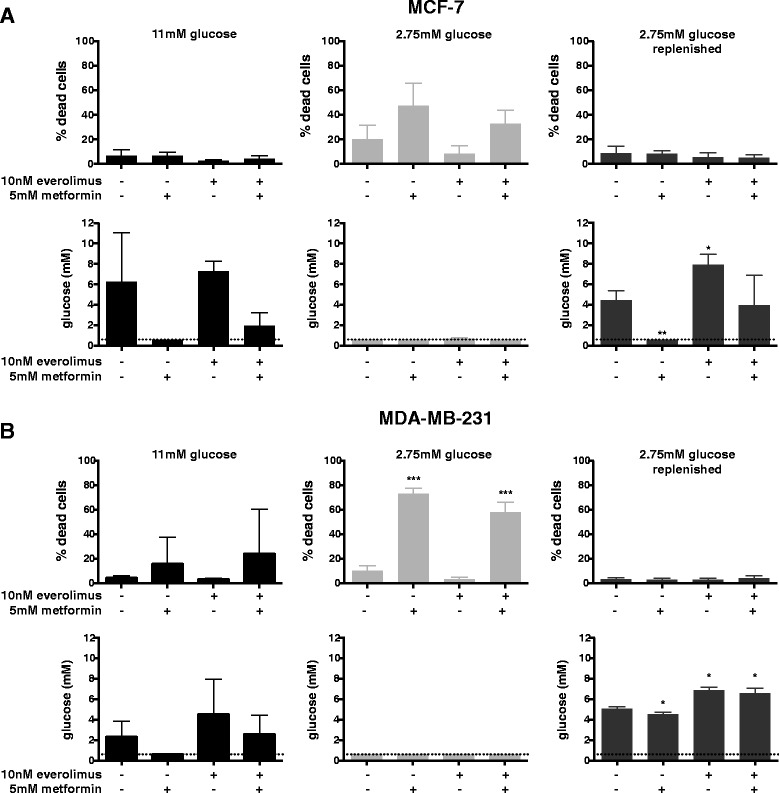
Metformin can only induce cell death under glucose-deprived conditions. MCF-7 (**a**) and MDA-MB-231 (**b**) cells were plated in medium containing 11 mM or 2.75 mM glucose and were treated with indicated concentrations of metformin and everolimus for 4 days. In addition, cells plated in 2.75 mM glucose were also replenished with 2.75 mM glucose every day (2.75 mM glucose replenished). AnnexinV/PI staining was used to quantify the percentage of dead cells after 4 days. Glucose concentration in the culture medium was determined after 4 days. The dashed line indicates the detection limit of the glucose meter. Data are presented as mean ± SD of three different experiments. Treated samples were compared to the same glucose concentration control. * *p* < 0.05; ** *p* < 0.01; *** *p* < 0.001

Because metformin increases glycolysis, as measured by ECAR, we predicted the glucose requirements of metformin-treated cells to be elevated. This high glucose demand may lead to glucose starvation in the in vitro setting and ultimately to apoptosis-mediated cell death. To quantify cell death, MCF7 and MDA-MB-231 cells were stained with annexin V and PI to determine cell death. MCF7 and MDA-MB-231 cells were plated and treated with everolimus or metformin for 4 days in media containing either 11 mM or 2.75 mM glucose. In the presence of metformin, cell death increased from 20 to 50% and from 10 to 70% in MCF7 and MDA-MB-231 cells, respectively, when cells were cultured in media containing 2.75 mM glucose. No metformin-induced effect on cell death was observed in media containing 11 mM glucose. Everolimus treatment did not induce cell death under any of the tested conditions (Fig. 5a-b).

To gain further insight in these glucose concentration-dependent cell death induced by metformin, we monitored glucose concentrations in culture media in time. The initial glucose concentration of 11 mM dropped faster following treatment of MCF7 cells with 5 mM metformin compared to no metformin (Additional file 2: Figure S1), which is in agreement with enhanced ECAR. The enhancing effect of metformin treatment on glucose consumption was less evident with MDA-MB231 cells. After 3 days of metformin treatment glucose was still detectable in culture media of both cell lines. In media with 2.75 mM glucose, levels dropped below 0.6 mM after 1 to 3 days. The fastest reduction in glucose levels below 0.6 mM was observed in media from cells treated with metformin, which is in line with the observed cell death (Additional file 2: Figure S1, Fig. 5a-b). In an additional set up, cells were therefore plated in 2.75 mM glucose containing media and each day supplemented with 2.75 mM glucose. Glucose concentrations were effectively kept above 0.6 mM during the 4 days metformin treatment, and the treatment did not result in cell death (Additional file 2: Figure S1, Fig. 5a-b).

These results demonstrated that metformin-induced cell death of MCF7 and MDA-MB-231 cells was due to glucose exhaustion and not due to low glucose culture conditions per se.

### Hypoxia does not influence the efficacy of everolimus and metformin during long-term treatment

**Fig. 6:**
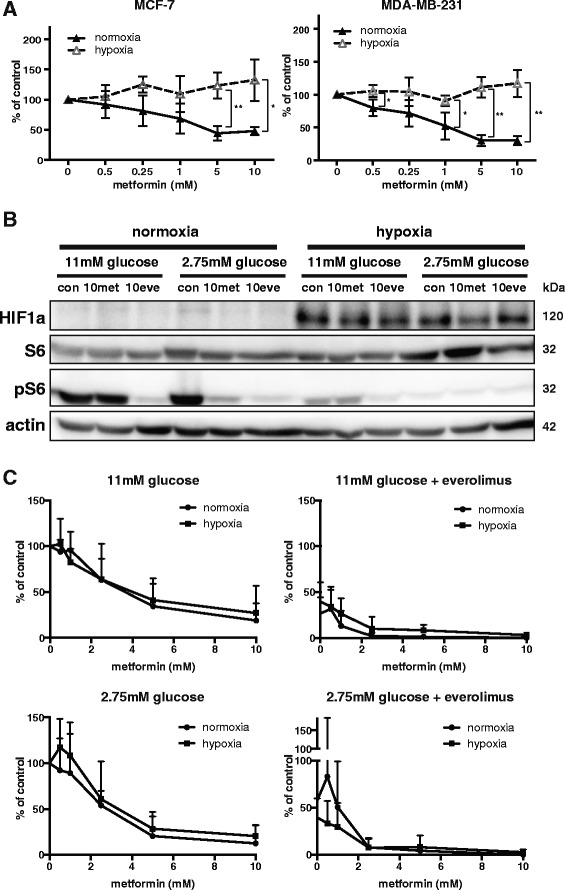
Hypoxia affects the efficacy of metformin only during short-term experiments. **a** MCF-7 and MDA-MB-231 cultured in 11 mM or 2.75 mM glucose-containing medium were treated with indicated concentrations of everolimus and metformin under normoxic or hypoxic conditions. Glucose was not replenished throughout the treatment. Cell viability was measured after 96 h using an MTT assay. Hypoxia alone already decreased cell viability to 50% of the viability in normoxia. Data are presented as mean ± SD of three different experiments. * *p* < 0.05; ** *p* < 0.01; *** *p* < 0.001. **b** MCF-7 cells cultured in 11 mM or 2.75 mM glucose-containing medium were treated with indicated concentrations of everolimus and metformin under normoxic or hypoxic conditions for 48 h. Western Blotting was carried out for HIF1a, S6 and p-S6. A representative blot of 2 experiments is shown. **c** MCF-7 cells were plated in medium containing 11 mM or 2.75 mM glucose at a concentration of 500 cells/well. After 8 days of treatment, MCF7 colonies, consisting of at least 50 cells, were counted. MCF7 cells were treated with indicated concentrations of metformin +/− everolimus (10 nM). Data are presented as mean + SD of three different experiments

Hypoxia is often observed in tumors. Under hypoxic conditions mitochondrial respiration and ATP production are compromised and cells are forced to use glycolysis, which may reduce the efficacy of metformin as mitochondrial inhibitor in these cells. Since hypoxia and low glucose concentrations have been found to co-occur in the same regions of a tumor [18], we tested the efficacy of metformin on cells cultured in 2.75 mM glucose combined with hypoxia. Hypoxia only modestly affected cell survival compared to survival under normoxic conditions, indicating that cells were still proliferating. However, effect of metformin on survival was lost in hypoxic, low glucose conditions in a short-term assay (Fig. 6a). Western Blotting of hypoxia inducible factor 1 α (HIF1α) confirmed hypoxic culture conditions. Cellular p-S6 levels in hypoxic conditions were reduced in 11 mM glucose and almost lost in 2.75 mM glucose containing media (Fig. 6b). Because the effect of hypoxia on p-S6 was so strong in 2.75 mM glucose containing media, no further reduction in p-S6 with everolimus or metformin treatment could be visualized.

### Glucose concentration and hypoxia do not affect inhibition of colony formation by everolimus and metformin

Next, we investigated the relationship between colony forming capacity and glucose concentration, hypoxia and inhibitory effects of everolimus and metformin. MCF7 cells were used in this long-term assay, since these cells developed easy measurable colonies. Everolimus and metformin both inhibited colony formation in a concentration dependent manner (Fig. 6c and Additional file 3: Figure S2). The glucose concentration in clonogenic assay media had no effect on the colony forming capacity of the cells or the inhibitory effects of everolimus and metformin. Replenishment with 2.75 mM glucose at a 2-day interval to prevent glucose shortage during the course of this long-term experiment did not change the results (Additional file 3: Figure S2). This is in agreement with the observation that the glucose concentration of the medium did not change considerably within the course of the clonogenic experiment at any condition (data not shown). Colony formation was also not affected by hypoxia. Moreover, efficacy of everolimus and metformin was similar in normoxia and hypoxia, both in low and high glucose conditions in the clonogenic assay (Fig. 6c). These results explain why the combination of everolimus and metformin had a strongly additive inhibitory effect on colony formation under all circumstances (Fig. 6c and Additional file 3: Figure S2).

## Discussion

In the present study, we show that everolimus and metformin both inhibit mTOR activity and have additive inhibitory effects on glucose metabolism, tumor cell growth and colony formation. These effects are evident in high and low glucose conditions and not reduced in the presence of hypoxia. These results support further in vivo investigation of everolimus combined with metformin as a putative anti-cancer therapy.

We found that the inhibitory effects of metformin on growth and colony formation of breast cancer cells were additive to the effects of everolimus in high and low glucose conditions, even when relatively low concentrations of both drugs were used. A previous study with different mutant p53 breast cancer cell lines cultured in high glucose media, demonstrated efficacy of metformin even at lower concentrations in both MTT and mammosphere assays, while higher concentrations of everolimus were required compared to our study [19]. Wang et al. also showed in vivo efficacy of the combination in xenograft bearing mice. Metformin sensitivity has been related to the presence of mutant p53 [20] and everolimus sensitivity to the presence of wild type p53 [21]. In our cell line panel, everolimus was indeed effective in wild-type p53 cells (MCF7) and less effective in mutant p53 cells (MDA-MB231 and T47D), but the preferential sensitivity of metformin in mutant p53 cells was not observed. Thus, more studies are required to investigate metformin and everolimus sensitivity in relation to the p53 status in breast cancer models. Interestingly, the combination of everolimus and metformin effectively inhibited colony and mammosphere forming capacity of wild type and mutant p53 breast cancer cells [Fig. 6c], [19]. These results suggest that tumor initiating cells are also sensitive to this combination in addition to bulk tumor cells as measured in the MTT assay, making this combination even more attractive to be further explored in breast cancer.

The inhibitory effect on mTOR has been described for each drug individually [22]. Here, we demonstrate that mTOR signaling is additively inhibited by the combination treatment. For everolimus it was expected that reduced mTOR activation would lead to reduced transcription of glycolytic enzymes and therefore a shift to mitochondrial respiration [23]. In contrast, we observed that everolimus inhibits mitochondrial respiration, which was not compensated by an enhanced glycolysis rate, as previously reported for hepatocellular carcinoma cells as well [24]. In human pancreatic cancer cell lines, however, everolimus treatment reduced the rate of glycolysis. Unfortunately, the effect on mitochondrial respiration was not reported [25]. At a mechanistic level, it has been shown that mTOR stimulates translation of mitochondrial mRNAs by inhibiting 4EBPs [26]. Consequently, mTORC1 inhibition leads to less mitochondrial mRNA translation and less mitochondrial respiration, which is in agreement with our results that everolimus treatment resulted in reduced p-S6 levels and mitochondrial respiration in both MCF7 and MDA-MB231 cells. As expected treatment with the mitochondrial complex I inhibitor metformin inhibited mitochondrial respiration and induced glycolysis [27–29]). Metformin treated cells had also a reduced maximal respiratory capacity. This might be an indirect effect of the reduced mTORC1 activity caused by metformin treatment. The additive metabolic effect of everolimus combined with metformin has not been described before. Our metabolic measurements show that a combination of both drugs, even at relatively low concentrations, completely reduced p-S6 and disrupted mitochondrial respiration. Moreover, similar results were obtained in high and low glucose containing media.

We demonstrate that metformin can induce a metabolic shift to increased glycolysis. The increased glucose utilization in the presence of metformin, especially under low glucose conditions, results in an earlier onset of glucose starvation and more cell death in MCF7 and MDA-MB231 breast cancer cells. This finding is in accordance with previous reports using glucose-free culture conditions [28, 30–32]. Here, we show that under stable low glucose conditions, thus preventing glucose starvation by replenishment of glucose, metformin treatment still results in growth inhibition, but cell death does not occur. Since glucose concentrations are relatively stable in vivo, our model under stable low glucose conditions in this respect is likely to reflect the situation in tumors in vivo [33, 34]. This is indirectly supported by in vivo observations showing that tumors from metformin treated patients did not have increased numbers of apoptotic cells compared to placebo treated patients [35]. Previous in vitro studies in breast cancer cell line models identified apoptosis as a mechanism of metformin’s anti-cancer effects. Unfortunately, glucose concentrations were not measured [22, 36, 37]. Our results strongly suggest that these findings were likely to be caused by in vitro glucose starvation due to metformin-induced increases in glucose utilization.

Other tumor-microenvironmental factors such as intratumoral pH, glutamine concentration, and oxygen tension are likely to influence the in vivo efficacy of metformin [38, 39]. Like metformin treatment, hypoxia shifts cellular metabolism towards glycolysis. This effect could either lead to synergy or reduce metformin effects, as shown in a sarcoma cell line model [39]. However, the sarcoma cell line study CoCl_2_ was used as a hypoxia mimetic in a short-term assay. We demonstrate that although in in vitro short-term experiments the effects of metformin in hypoxic conditions appeared to be reduced, longer-term assays showed that metformin activity is maintained. In the short-term setting, hypoxia alone already decreased cell proliferation, so that possible effects of metformin on proliferation were harder to detect. Indeed, we found that p-S6 had almost completely disappeared under hypoxic, low glucose conditions suggesting that these cells were not actively proliferating. During the long-term clonogenic assay, cells sufficiently adapted to hypoxia and formed colonies. Metformin was effective against these colonies under hypoxic conditions, suggesting that metformin will retain its efficacy in the hypoxic areas of an in vivo tumor.

Blood plasma levels of metformin in patients (around 0.05 mM) are at least twenty-fold lower than those used in most in vitro cell line studies (1–40 mM) [40]. Although there is evidence that the acidophilic properties of metformin may cause accumulation in the mitochondrial matrix, thereby decreasing the systemic levels required, the low concentrations which potentiate everolimus in our model are promising [41].

Treatment of cancer cells with inhibitors of both glycolysis and mitochondrial respiration, leading to synthetic metabolic lethality is a promising therapeutic strategy to kill cancer cells. Combination of metformin with the glycolytic inhibitor dichloroacetate effectively induced cell death in breast and ovarian cancer cell lines [27]. Also in vivo combination treatment with the mitochondrial inhibitor phenformin and the lactate dehydrogenase inhibitor oxamate has been successful [42]. Combining metformin with everolimus is a novel example of dual metabolic targeting.

## Conclusions

Metformin and everolimus had additive inhibitory effects on both proliferation and colony formation in vitro in breast cancer cell line models at high and low glucose concentrations in normoxia and hypoxia. Mechanistically, mTOR inhibition and dual metabolic targeting appear important. Metformin can be used to prevent and/or treat everolimus-induced hyperglycemia, while potentially enhancing the anti-cancer effects of everolimus.

## Additional files

Additional file 1: Figure S3. Uncoupling of the mitochondrial respiration with FCCP after treatment with metformin and everolimus for 48 h. Using the seahorse XF analyzer, the basal OCR and maximal OCR after uncoupling of mitochondrial respiration with FCCP of MCF-7 (A) and MDA-MB-231 cells (B) in response to 48 h of metformin or everolimus treatment was determined. Basal OCR in untreated cells was set at 100% as a reference to which all other mitochondrial respiration values of the same glucose concentration group were correlated. Data are presented as mean ± SD of three different experiments. (PDF 69 kb)

Additional file 2: Figure S1. Glucose concentration of cell culture medium during 4 days of metformin treatment. MCF-7 and MDA-MB-231 cells were plated at a concentration of 30.000 (MCF-7) or 80.000 (MDA-MB-231) cells in medium containing 11 mM or 2.75 mM glucose. Additionally, cells were plated in medium containing 2.75 mM glucose and replenished with 2.75 mM glucose every 24 h (2.75 mM glucose replenished). Cells were treated with 5 mM metformin for 4 days. 20 μl medium samples were taken for glucose concentration measurements every day. In the glucose-supplemented condition, this was done after addition of glucose. Data are presented as mean ± SD of three different experiments. (PDF 35 kb)

Additional file 3: Figure S2. Metformin and everolimus inhibit colony formation of breast cancer cell lines independently of glucose concentration. MCF-7 cells were plated in medium containing 11 mM or 2.75 mM glucose at a concentration of 500 cells/well. A subset of cells plated in 2.75 mM glucose was also replenished with 2.75 mM glucose every 48 h (2.75 mM glucose replenished). Cells were treated with indicated concentrations of everolimus and metformin for 8 days and colonies were counted. Data are presented as mean ± SD of three different experiments. (PDF 33 kb)

## Abbreviations

4EBP1: Eukaryotic initiation factor 4B binding protein
AMPK: AMP-activated kinase
ECAR: Extracellular acidification rate
FCCP: Carbonyl cyanide 4-(trifluoromethoxy)phenylhydrazone
FCS: Fetal calf serum
HIF1α: Hypoxia inducible factor 1 α
mTOR: Mammalian target of rapamycin
mTORC1: mTOR complex 1
mTORC2: mTOR complex 2
OCR: Oxygen consumption rate
S6: S6 ribosomal protein
SD: Standard deviation
