# Supplementary figures and images for: Anti-tumor effects of everolimus and metformin are complementary and glucose-dependent in breast cancer cells
Source: BMC Cancer. 2017 Mar 29;17:232. doi: 10.1186/s12885-017-3230-8 (PMC5372253; doi:10.1186/s12885-017-3230-8)

**MCF-7 11mM glucose**

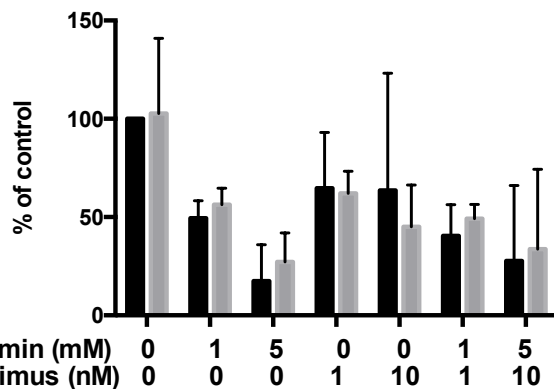

**MCF-7 2.75mM glucose**

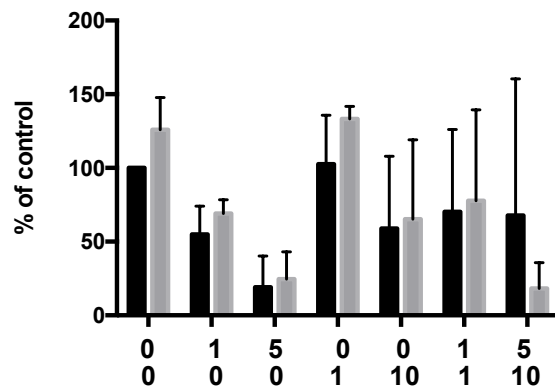

**MDA-MB-231 11mM glucose**

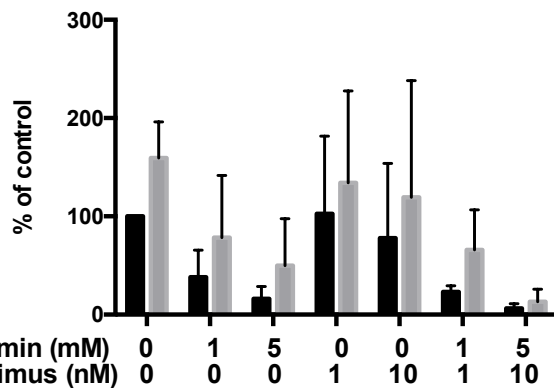

**MDA-MB-231 2.75mM glucose**

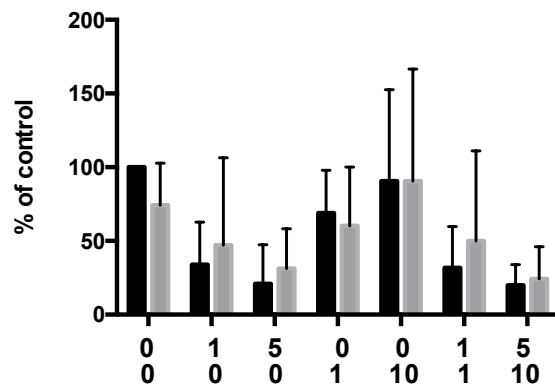

■ basal OCR

■ maximal OCR

Supplement: Supplementary file 1 — Uncoupling of the mitochondrial respiration with FCCP after treatment with metformin and everolimus for 48 h. Using the seahorse XF analyzer, the basal OCR and maximal OCR after uncoupling of mitochondrial respiration with FCCP of MCF-7 (A) and MDA-MB-231 cells (B) in response to 48 h of metformin or everolimus treatment was determined. Basal OCR in untreated cells was set at 100% as a reference to which all other mitochondrial respiration values of the same glucose concentration group were correlated. Data are presented as mean ± SD of three different experiments. (PDF 69 kb) [file 12885_2017_3230_MOESM1_ESM.pdf]

11mM glucose

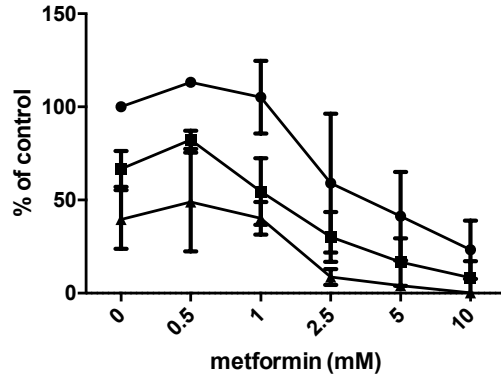

2.75mM glucose

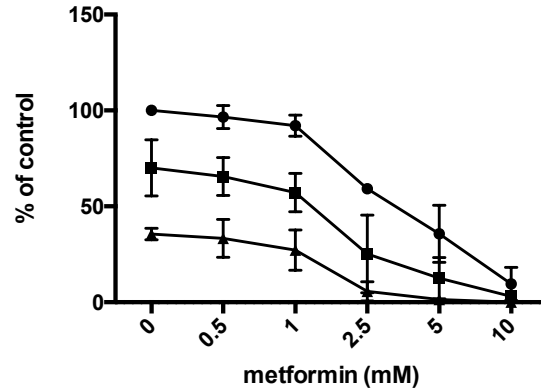

2.75mM glucose replenished

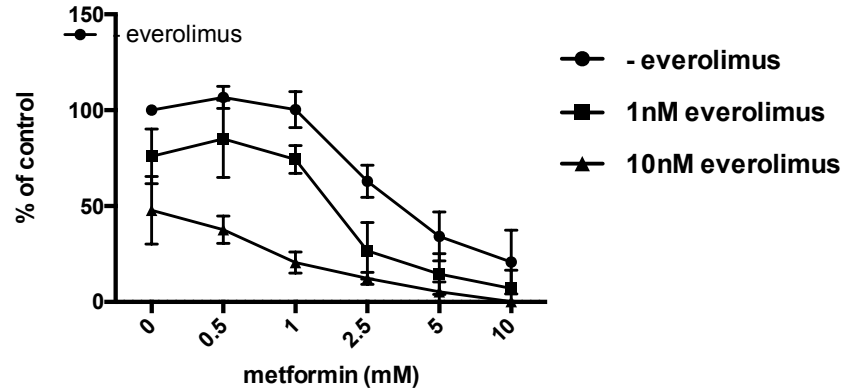

Supplement: Supplementary file 3 — Metformin and everolimus inhibit colony formation of breast cancer cell lines independently of glucose concentration. MCF-7 cells were plated in medium containing 11 mM or 2.75 mM glucose at a concentration of 500 cells/well. A subset of cells plated in 2.75 mM glucose was also replenished with 2.75 mM glucose every 48 h (2.75 mM glucose replenished). Cells were treated with indicated concentrations of everolimus and metformin for 8 days and colonies were counted. Data are presented as mean ± SD of three different experiments. (PDF 33 kb) [file 12885_2017_3230_MOESM3_ESM.pdf]
