# Supplementary material for: Anti-tumor effects of everolimus and metformin are complementary and glucose-dependent in breast cancer cells
Source: BMC Cancer. 2017 Mar 29;17:232. doi: 10.1186/s12885-017-3230-8 (PMC5372253; doi:10.1186/s12885-017-3230-8)
Supplement: Supplementary file 2 — Glucose concentration of cell culture medium during 4 days of metformin treatment. MCF-7 and MDA-MB-231 cells were plated at a concentration of 30.000 (MCF-7) or 80.000 (MDA-MB-231) cells in medium containing 11 mM or 2.75 mM glucose. Additionally, cells were plated in medium containing 2.75 mM glucose and replenished with 2.75 mM glucose every 24 h (2.75 mM glucose replenished). Cells were treated with 5 mM metformin for 4 days. 20 μl medium samples were taken for glucose concentration measurements every day. In the glucose-supplemented condition, this was done after addition of glucose. Data are presented as mean ± SD of three different experiments. (PDF 35 kb) [file 12885_2017_3230_MOESM2_ESM.pdf]

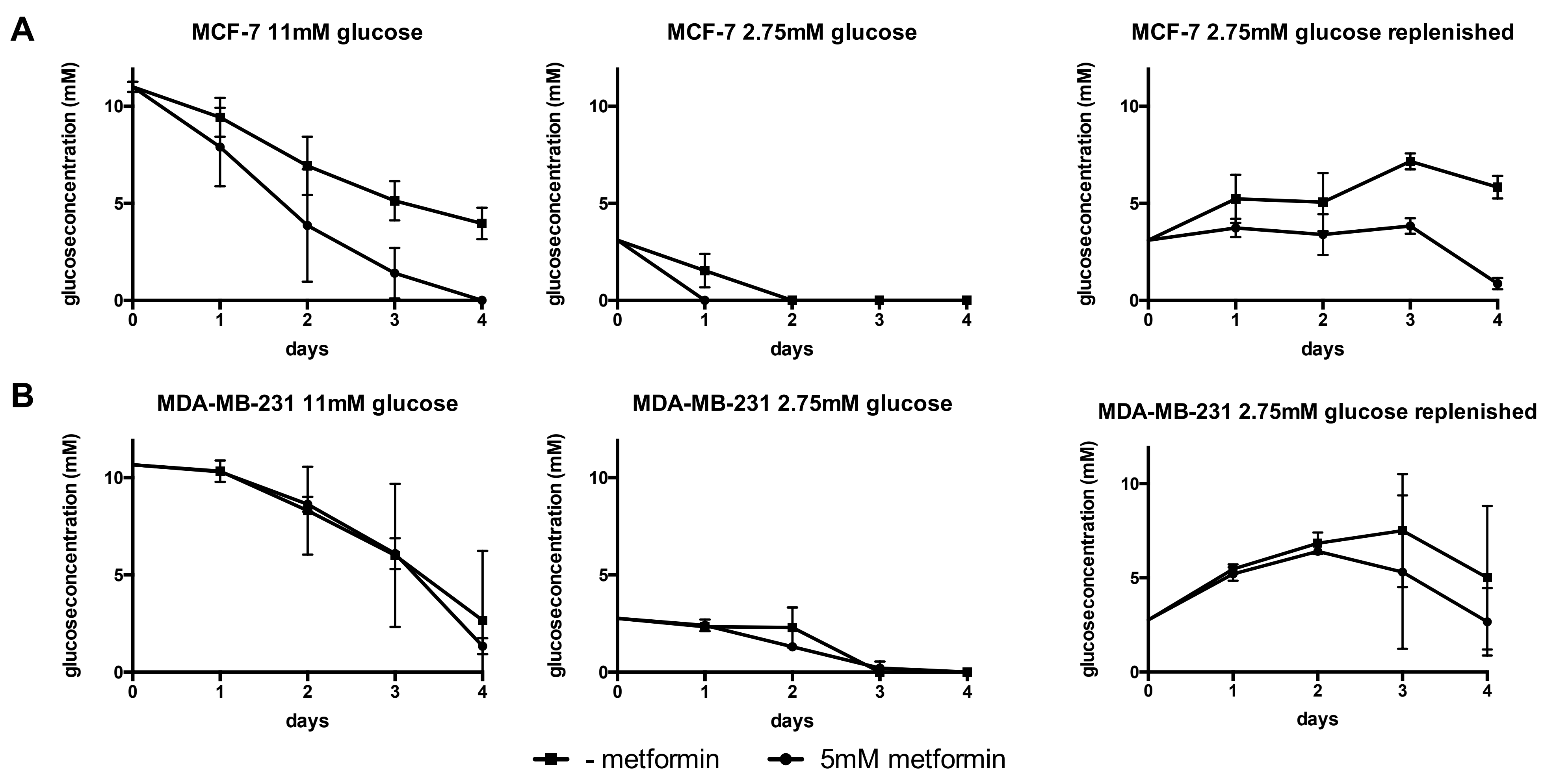
A
MCF-7 11mM glucose
MCF-7 2.75mM glucose
MCF-7 2.75mM glucose replenished
B
MDA-MB-231 11mM glucose
MDA-MB-231 2.75mM glucose
MDA-MB-231 2.75mM glucose replenished
glucoseconcentration (mM)
days
0
5
10
1
2
3
4
- metformin
5mM metformin
